# Chemical Characterization of Polysaccharide Extracts Obtained from Pomace By-Products of Different White Grape Varieties

**DOI:** 10.3390/molecules28196770

**Published:** 2023-09-22

**Authors:** María Curiel-Fernández, Marta Bueno-Herrera, Zenaida Guadalupe, Belén Ayestarán, Silvia Pérez-Magariño

**Affiliations:** 1 Grupo de Enología, Instituto Tecnológico Agrario de Castilla y León, Ctra Burgos Km 119, 47071 Valladolid, Spain; curferma@itacyl.es (M.C.-F.);; 2 Departamento de Agricultura y Alimentación, Instituto de Ciencias de la Vid y el Vino (Universidad de La Rioja, Gobierno de La Rioja, CSIC), Finca de La Grajera, Ctra. Burgos 6, 26007 Logroño, Spain; zenaida.guadalupe@unirioja.es (Z.G.); belen.ayestaran@unirioja.es (B.A.)

**Keywords:** revalorization, grape pomace, by-products, polysaccharides, bioactive compounds, grape variety

## Abstract

Grape pomace is one of the main by-products in the wine industry and contains some high-added-value compounds, such as polysaccharides. Considering the wide application possibilities of polysaccharides in wine and in the food industry, the revalorization of grape pomace to extract polysaccharides presents itself as an opportunity for by-product management. Therefore, the aim of this study was to characterize polysaccharide extracts obtained from pomace by-products of different white grape varieties. The type and content of polysaccharides, proteins and phenols were analyzed. Statistically significant differences were found between the varietal extracts in the types and concentrations of polysaccharides. The extracts obtained from the *Verdejo* and *Puesta en Cruz* varieties showed the highest polysaccharide purity and contents, but the type of polysaccharides was different in each case. The *Verdejo* provided extracts richer in non-pectic polysaccharides, while the *Puesta en Cruz* provided extracts richer in pectic polysaccharides. The protein and polyphenol contents were low in all extracts, below 2.5% and 3.7%, respectively. These results open up a new possibility for the revalorization of grape pomace by-products to obtain polysaccharide-rich extracts, although it would be interesting to improve both the yield and the purity of the extracts obtained by studying other extraction techniques or processes.

## 1. Introduction

The wine industry generates different types of waste throughout the winemaking process involving organic and inorganic wastes. Organic wastes mainly include stems, grape pomace and lees [1]. Grape pomace is the by-product remaining after the pressing process and accounts for approximately 20–30% (in fresh weight) of the grapes used for the winemaking process [2,3]. Grape pomace consists of grape skins, pieces of stems, seeds and sometimes residual pulp [4]. Different factors can determine the amount of grape pomace obtained, such as grape variety and the techniques used in winemaking, especially the pressing process [5].

These solid residues are often used for distillation, tartaric acid production, as fertilizers, as animal feed, as compost and as biomass for energy production [3,6,7]. However, grape pomace also contains some high-value-added compounds, such as polyphenols, polysaccharides, fibers, proteins, minerals and seed oil that offer an important possibility for its revalorization [5,8]. The most studied bioactive compounds are polyphenols because of their antioxidant, antimicrobial and anti-inflammatory properties and their benefits to the cardiovascular system [1,2,9]. Polysaccharides present in grape skins have an essential protective role and mechanical resistance function due to their structural role played mainly by pectins, celluloses and hemicelluloses [10]. Throughout the winemaking process, the interaction between grape polysaccharides and other compounds in the wine depends on the structure of the polysaccharides [10]. Polysaccharides contained in the grape cell wall can be polysaccharides rich in arabinose and galactose (PRAG), which contain arabinans (A), arabinogalactans (AG), arabinogalactan proteins (AGP), rhamnogalacturonans types I and II (RG-I and RG-II), homogalacturonans (HG) and non-pectic polysaccharides (NPP), such as hemicelluloses, celluloses and mannans [10]. Some factors can influence the composition and content of grape polysaccharides, such as terroir, ripeness and grape variety [11,12,13]. Polysaccharides play an important role in the winemaking process because they act as protective colloids [14] and have technological functions in wine, such as preventing tartrate crystallization and improving protein stability [15,16]. Moreover, polysaccharides have an influence on the sensorial characteristics of wines, improving the color stability in red wines [17], modifying the volatility of aromatic compounds [18] and increasing the full-body perception of wines [19,20].

Currently, the revalorization of wine by-products is also related to the circular economy, i.e., it focuses on obtaining bioactive compounds from food by-products and their subsequent application in the winemaking process. Therefore, several authors have studied the extraction of polysaccharides from food by-products due to their wide applications in the food industry as a natural food additive. Polysaccharides, and especially pectic polysaccharides, are used in the food industry as emulsifiers, thickeners, stabilizers, gelling agents and texture modifiers [21]. In addition, fruit by-products and fruit pomaces are the main materials studied to improve pectin yields [22,23,24]. Polysaccharides are also of interest in the pharmaceutic industry because of their potential antioxidant and anticarcinogenic activities [25].

Considering the wide application possibilities of polysaccharides in the winemaking process, and also in different processes of the food industry, the revalorization of grape pomace and the extraction of polysaccharides present an opportunity for by-product management. Therefore, the aim of this study was to characterize the extracts rich in polysaccharides obtained from grape pomaces of different white grape varieties in order to valorize these by-products. The type of polysaccharides, their content and the distribution of molecular weights were determined. In addition, other possible compounds that could be present in the extracts were also analyzed, such as proteins, polyphenols and monomeric phenolic compounds.

## 2. Results and Discussion

### 2.1. Oenological Parameters of Grapes

Table 1 shows the oenological parameters of the varietal grapes used to obtain the different extracts from their pomace. The harvested moment of the grape varieties was decided by each winegrower or oenologist, and, therefore, the grapes presented differences in the degree of maturation. The Brix degree ranged from 18.2 to 24.2, with the LIG being the variety with the highest Brix degree followed by the VER1 and MALV. The grape varieties with the lowest Brix degree were the VIU, PC and AM. The titratable acidity also showed high differences between the varietal grapes and most of them varied from 5.0 to 7.2 g/L. Only the SB showed a very high acidity value (10.2 g/L). Both Verdejo samples showed differences in the degree of ripening, with the VER1 with a higher degree (23.4 °Brix) than the VER2 (21.2 °Brix). 

### 2.2. Monosaccharide Composition and Polysaccharide Families of Grape Pomace Extracts

Table 2 shows the monosaccharide composition and polysaccharide families of each varietal polysaccharide extract. Glucose is the monosaccharide with the highest amount in all varietal extracts, according to previous results obtained by other authors in pomace and cell wall materials from white grape pomace [26,27,28,29]. The VER1 extracts presented the highest content of glucose, with a content of 271 mg/g, representing 82% of the total monosaccharides (TMS) analyzed, followed by the RSB and VIU extracts, with the same content of 172 mg/g, representing 74% and 70% of TMS, respectively. The same differences were observed in the content of non-pectic polysaccharides (NPP) among the varietal extracts due to glucose being the main component. These results agree with those obtained by Canalejo et al. [29]. Vidal et al. [19] observed that glucose is the main glycosyl-residue present in the cell walls of grape pulp and skin and is the major component of the main structural polysaccharides of the grape cell walls, such as cellulose, hemicelluloses or xyloglucans, arabinoglucans and mannans. The next highest content of monosaccharides was galactose and galacturonic acid, followed by rhamnose and arabinose in all varietal extracts. These monosaccharides are constituents of pectic polysaccharides. The galacturonic acid content was used to estimate the homogalacturonans (HG), and the contents of galactose, arabinose, rhamnose and glucuronic acid were used to estimate the pectic polysaccharides rich in arabinose and galactose (PRAG). The minority glycosyl residues in all varietal extracts were xylose, mannose, fucose, 2-*O*-methyl xylose, 2-*O*-methyl fucose, apiose and KDO. Mannose, xylose and fucose in grapes are associated with the presence of hemicelluloses [30]. The xylose residues were components of xyloglucans [31,32], and mannose was attributed to mannans and hemicellulose from grape pericarp [33,34]. The presence of minor carbohydrates, 2-*O*-methyl xylose, 2-*O*-methyl fucose, apiose and KDO were used to estimate RG-II content, considering also rhamnosyl, arabinosyl, galactosyl and galacturonosyl residues [35].

Total monosaccharides (TMS) were calculated as the sum of the monosaccharide compounds of each varietal extract. Statistically significant differences in the TMS content of the extracts studied by grape variety were found. The VER1 and PC extracts were the varieties with the highest content in TMS, with a value of 330 and 325 mg/g, respectively. On the other hand, the LIG and MALV extracts presented the lowest content in TMS, with 144 and 141 mg/g, respectively.

The remaining varietal extracts showed an average TMS content of 230 mg/g. These results were similar to those obtained by González-Centeno et al. [30] in white grape pomace and slightly lower than those obtained by Canalejo et al. [35] and Pérez-Magariño et al. [36] in extracts from grape pomace of the Viura and Verdejo variety, respectively, and Apolinar-Valiente et al. [13] from red grape marc of the Monastrell variety.

The VER1 extracts showed a higher content of NPP, which are structural polysaccharides, and a lower content of major pectic polysaccharides (HG and PRAG) than the VER2 extracts. This fact could be associated with the degree of ripening of each Verdejo grape since the VER1 grapes presented a higher Brix degree than the VER2 grapes. Gao et al. [37] observed a greater degradation of skin cell wall pectin than of the structural polysaccharides in more mature grapes. Probably, the higher extraction by pressing pectic polysaccharides from the skin to the must of VER1 resulted in the lower content of HG and PRAG in the grape pomace extracts of VER1 compared to VER2. However, the RG-II content was similar in both extracts (VER1 and VER2). In general, the RG-II content of the varietal extracts of this study was lower than those reported by Canalejo et al. [35] in extracts from the Viura grape variety.

The Brix degree of the PC grapes was lower than that of the LIG, VER1 and MALV grape varieties, but similar to that of the AM and VIU varieties. The higher degree of ripening of the LIG, VER1 and MALV varieties explains the lower content of HG, PRAG and RG-II in these extracts than in the PC extracts, with the exception of RG-II in the MALV and VER1 extracts, which was similar to that of the PC extracts. At a lower degree of ripening, the cell walls are more intact and present greater resistance to the extraction of cell wall polysaccharides from the skin [38]; consequently, the PC extract was richer in pectic polysaccharides. However, only the effect of the ripening degree cannot explain the lower content of pectic polysaccharides (HG, PRAG and RG-II) and mannans in the VIU extracts with respect to the PC extracts from grape varieties with a similar Brix degree (Table 1). The AM and PC extracts obtained from grape varieties with a similar Brix degree presented similar values of pectic polysaccharides, except for HG and MN, with the AM extracts showing lower values of HG and higher values of MN than the PC extracts (Table 2). This is probably due to the fact that the physicochemical and biochemical characteristics of the cell walls of the VIU variety were more different than those of the PC and AM varieties [39]. Therefore, the lower cell wall thickness of the VIU grapes explains the greater extraction of pectic polysaccharides and mannans to the must by pressing, leaving a pomace less rich in polysaccharides. The extracts obtained from the RSB, SB and VER2 varieties with a slightly higher Brix degree than the PC variety (Table 1) also had a lower pectic polysaccharide content than the PC variety. These results indicate that the PC variety harvested at a low degree of ripening was the most suitable for obtaining pomace extracts with a higher pectic polysaccharide content (TPP). This variety is probably characterized by a thicker cell wall, which hindered the extraction of polysaccharides from the skin to the must, resulting in pomace richer in pectic polysaccharides. To our knowledge, there are no studies on the cell wall structure of the PC variety or of the other grape varieties studied in this work.

Three ratios were calculated to estimate the different sugar structures contained in the varietal extracts. The Ara/Gal ratio shows the characteristic structures of PRAGs and their richness in arabinose [40]. In our study, the Ara/Gal ratio of the extracts obtained from the different varietal grape pomaces was less than 0.5, so these structures were richer in galactose than in arabinose. The Rha/GalA ratio provides information on the presence of homogalacturonan and rhamnogalacturonan structures [33]. The resulting low values of this ratio, below 0.5, are due to the lower content of rhamnose than galacturonic acid, indicating the presence of more homogalacturonan-type structures.

Finally, the ratio (Ara + Gal)/Rha estimates the relative importance of the neutral side chains in the rhamnogalacturonan backbone since most of the galactose and arabinose content is associated with the pectin hairy regions [13]. The results obtained showed differences between the varietal extracts, with those from the MALV and AM varieties having the highest values of this ratio, suggesting that the rhamnogalacturonan chains have more neutral side chains compared to the rest of the varieties. The observed differences agree with those observed by Apolinar-Valiente et al. [41].

### 2.3. Molecular Weight Distributions of Polysaccharides of Grape Pomace Extracts

**Figure 1:**
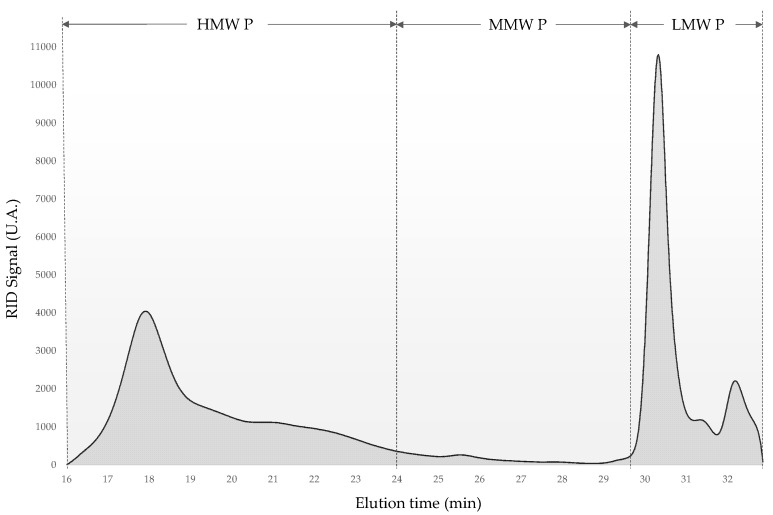
HPSEC-RID chromatogram of the extract from Albillo Mayor grape pomace and the three different polysaccharide fractions by molecular weight.

Figure 1 shows the qualitative changes in the molecular weight distribution of the varietal polysaccharide extracts. This chromatogram was the result of the HPSEC-RID analysis of the AM extract, and three different polysaccharide fractions by molecular weight are indicated. The polysaccharide molecular weight chromatograms of all extracts are shown in the Supplementary Material (Figure S1). High molecular weight polysaccharides (HMW P) are mainly related to PRAGs. Medium molecular weight polysaccharides (MMW P) include molecules of RG-II dimers. Finally, low molecular weight polysaccharides (LMW P) include oligosaccharides and low molecular weight fragments of larger molecules, such as AGP, MN and RG-II monomers [10].

The results of the HPSEC-RID analyses are shown in Table 3. The LMW P fraction represented the highest content followed by HMW P. As can be seen, there are statistically significant differences in the distribution of HMW P and LMW P. The MALV, PC and AM extracts presented higher values of HMW P than LMW P, with a range of 62.5% to 58.3%. On the contrary, the extracts obtained from the rest of the grape varieties studied showed a higher content of LMW P than of HMW P, with VER1 being the extract with the lowest content of HMW P with a value of 37.7%. In addition, only the AM extract presented MMW P contents with a low value of 1.4%.

The results obtained for the molecular weight distribution of both extracts of the Verdejo grape variety (VER1 and VER2) showed a high LMW P content and were similar to those obtained by Pérez-Magariño et al. [36] in polysaccharide extracts obtained from the Verdejo grape pomace. The HMW P values were higher than the content obtained by Canalejo et al. [29] in extracts from the Viura grape pomace.

### 2.4. Total Proteins, Total Polyphenols and Monomeric Phenolic Compounds of Grape Pomace Extracts

Proteins and phenolic compounds are other macromolecules present in the cell walls of grape skins. Proteins are found in the matrix formed by structural polysaccharides and play an important role because they act as cross fibers that reinforce the mechanical resistance of the grape skin [10,42]. Furthermore, some proteins are bound to polysaccharides, such as AGPs, and, in this structure, there is a chain link with the proteins. On the other hand, phenolic compounds in grapes are found mainly in the solid parts, such as the skins and seeds. Therefore, these compounds could be present in the extracts obtained from grape pomace, although the extraction process is focused on obtaining extracts rich in polysaccharides.

Table 3 shows the total protein content of the different varietal extracts studied, which ranged from 14.0 to 24.5 mg BSA/g. Statistically significant differences were found between the varietal extracts. The LIG extracts showed the highest content, with a value of 24.5 mg/g. Nunan et al. [43] observed an increase in proteins throughout the ripening process, so riper grapes are expected to have a higher protein content. Therefore, the high protein content in the LIG extract could be associated with the higher Brix degree of this variety. On the contrary, the lowest values were presented by RSB and AM, with 14.5 and 14 mg BSA/g, respectively. The two extracts from the Verdejo variety did not show differences between them and have also a similar protein content to that of the SB, PC and MALV extracts. Moreover, the PC and MALV extracts presented a similar protein content to the LIG extract. Even so, in general, it can be considered that the total protein content of all the extracts studied was low at less than 2.5% of the extract. The values obtained were lower than those obtained by other authors in cell wall material isolated from red grape marc [13] or from fresh red grape skins [39], and slightly lower than those obtained by Canalejo et al. [29] in polysaccharide extracts from Viura white grape pomace. The observed differences between the extracts in terms of protein content could be associated with the grape variety, as other authors have found in previous studies [13,39].

Table 3 shows the obtained results in total polyphenols (TP) of the studied varietal extracts. As can be seen, statistically significant differences were found between the varietal extracts and the TP range was from 17.2 to 36.9 mg GA/g. The highest contents corresponded to the RSB and LIG extracts, with a content of 36.9 and 35.1 mg GA/g, respectively, and the lowest to the AM and MALV extracts, with a range of 17.2 and 19.6 mg GA/g, respectively. These values were lower than those obtained by Canalejo et al. [29] in polysaccharide extracts of Viura grape pomace or Tempranillo grape marc, as well as those obtained in cell wall material isolated from red grape marc or fresh red grape skins [13,39]. Considering the results obtained by other authors, the TP content could vary during the ripening process and with the grape variety [13,39,44], so both factors may influence the TP values of our extracts. However, as commented above for the total proteins, the TP content was low in these extracts, with a value of less than 3.7% of the extract.

Forty different individual phenolic compounds were tested, but only those listed in Table 4 were identified and analyzed. Statistical significative differences were found in all the quantified phenolic compounds by varietal extract. The extract of VER1 showed the highest content of all phenolic compounds, with the exception of gallic acid, which was much higher than the rest of the extracts. Flavanols were the most abundant compounds in all the varietal extracts, with catechin showing the highest values, ranging from 79.2 to 1172 µg/g, followed by epicatechin, with a range of 11.7 to 206 µg/g. All detected TE-HCA compounds were only found in the VER1 and VIU extracts, and t-caftaric acid was also found in the VER2 and AM extracts. These results were similar to or lower than those obtained by Canalejo et al. [29] in polysaccharide extracts of Viura grape pomace or Tempranillo grape marc. No further works have been found to evaluate the content of individual phenolic compounds in extracts. In general, the concentrations of these phenolic compounds were also very low at less than 0.3% of the extract.

These results may also suggest that the polysaccharide extraction process is specific and, therefore, the content of PR, TP and monomeric phenolic compounds in the extracts are low.

### 2.5. Yield and Polysaccharide Purity of the Grape Pomace Extracts

The yield of the grape pomace extracts was determined by the grams of lyophilized extract obtained per 100 g of dry-weight pomace. Statistically significant differences were found in the extract yields obtained that ranged from 5.54% to 15.9%, corresponding to PC and AM, respectively. Furthermore, the AM and VER1 extracts presented the highest yield, with values of 15.9% and 13.4%, respectively.

On the other hand, the polysaccharide purity of the obtained extracts was evaluated and was expressed as grams of TFP per 100 g of lyophilized extract. The polysaccharide purity was higher in the VER1 and PC extracts than in the others, with values of 32.9% and 31.6%, respectively. LIG and MALV were the extracts with the lowest polysaccharide purity, with values around 14%. In general, the polysaccharide purity of all varietal extracts was lower than that obtained by Canalejo et al. [30] in polysaccharide extracts obtained from Viura grape pomace.

### 2.6. Multivariate Statistical Analysis

**Figure 2:**
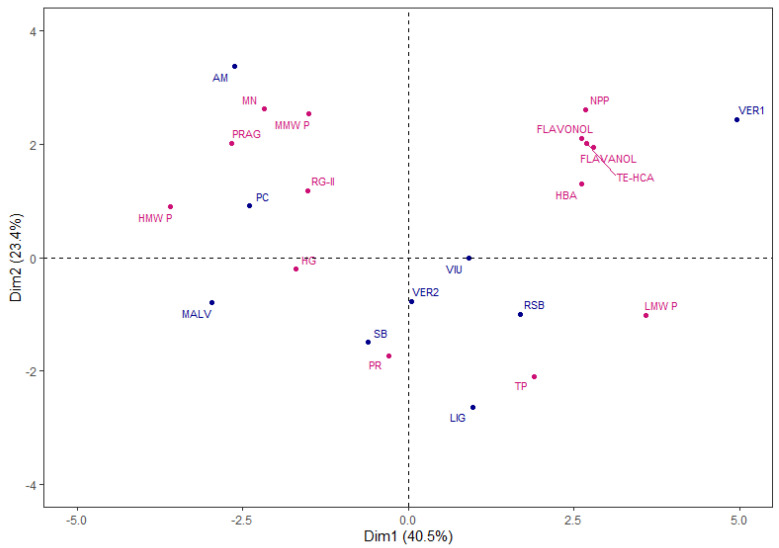
Principal component analysis. Representation of varietal extracts and variables used. Abbreviations of compounds and grape varieties in Materials and Methods section.

A principal component analysis (PCA) was performed to investigate the relationships between the variables as a whole and the varietal extracts studied. The PCA was performed with the following variables: molecular weight polysaccharides (HMW P, MMW P and LMW P), total protein and total polyphenol content (PR and TP), family polysaccharides (PRAG, RG-II, MN, HG and NPP) and monomeric phenolic compound families (HBA, TE-HCA, Flavanols and Flavonols). The PCA selected four components with an eigenvalue greater than one, which explained 90.2% of the total variance. Figure 2 shows the distribution of the varietal extracts in the plane defined by the first two dimensions that explained 63.9% of the total variance. As can be seen, the varietal extracts showed the differences between them associated with the different variables.

The VER1 extract, sited in the positive side of Dim 1 and 2, was clearly separated from the other extracts and was positively correlated with the HBA, HCA, Flavanols and Flavonols, as well as NPP and LMW P. On the other hand, the AM and PC extracts were located on the left side of the plane, correlating mainly with the remaining polysaccharide families, MN, PRAG, RG-II and HG, which correspond to the pectic polysaccharides. Finally, the remaining varietal extracts were located in the lower part of the plane and were more correlated with the PR and TP content.

Therefore, the grape variety will influence the chemical composition of the extracts obtained from each pomace.

## 3. Materials and Methods

### 3.1. Chemicals

Chromatographic-grade reagents were provided by Riedel-de-Haën (Honeywell, Germany). Water type I was obtained using Autwomatic Plus 1 + 2 GR equipment (Wasserlab, Barbatáin, Navarra, Spain, 2020).

Polysaccharide extractions were carried out with food-grade reagents, which are as follows: hydrochloric acid 37% (E-507, Panreac, Madrid, Spain), tartaric acid (E-334, Agrovin, Ciudad Real, Spain) and rectified ethanol from molasses 96 (0110F, Alcoholes Montplet, Barcelona, Spain). Polysaccharide standards (seven dextrans from 5 to 410 kDa and pectin), gallic acid, Coomasie Blue brilliant and Bovine Serum Albumin were supplied by Sigma-Aldrich (Steinheim, Germany).

The monosaccharide standards used to perform the calibration curves and phenolic compound standards were purchased from Fluka (Buchs, Switzerland), Sigma-Aldrich (Steinheim, Germany) and Extrasynthèse (Genay, France).

### 3.2. Grape Pomace Material

Nine grape pomace samples were obtained from eight different white grape varieties from the Castilla y León region located in northern Spain from the 2021 vintage. The grape varieties studied were as follows: Ligeruela var. (LIG), two different samples of Verdejo var. (VER1 and VER2), Malvasía var. (MALV), Sauvignon Blanc var. (SB), Rufete Serrano Blanco var. (RSB), Albillo Mayor var. (AM), Puesta en Cruz var. (PC) and Viura var. (VIU). LIG, PC and RSB are minority grape varieties from Castilla y León region.

The grapes were harvested at the optimum ripening degree determined by each winegrower or oenologist. After that, the grapes were destemmed, crushed, sulfited and pressed using a vertical hydraulic press in the Oenological Station placed in Rueda (Valladolid). The pressed grape pomaces were immediately frozen at −15 °C in airtight bags until their extraction.

### 3.3. Polysaccharide Extracts from Grape Pomaces

The polysaccharide extracts were obtained following the methodology previously developed by our group [35], with slight modifications. Briefly, grape pomaces were defrosted and homogenized with the Ultra Turrax T25 digital (IKA, Staufen im Breisgau, Germany, 2009). The extraction was carried out with 15 g of grape pomace in acidic conditions (2.5 g/L of tartaric acid at pH 1) in ultrasound bath (Pselecta, Barcelona, Spain, 2006) for 30 min followed by stirring in orbital shaker (Proeti, Madrid, Spain, 2018) for 18 h. Then, the samples were centrifuged (3500× *g* for 20 min, 4 °C), and the supernatant was concentrated five times using a rotary evaporator R-210 (Büchi, Switzerland, 2010). Then, the polysaccharides were precipitated with four volumes of acidified alcohol for 24 h at 4 °C. The precipitate was centrifuged (3500× *g* for 20 min, 4 °C) and the pellet was freeze-dried. All the extractions were carried out in triplicate.

### 3.4. Oenological Parameters of Grapes

Standard oenological parameters of grapes were determined according to the official methods of OIV [45]. Titratable acidity was determined with a Crison pH meter GLP22 (Barcelona, Spain, 2010) according to the method described in OIV-MA-AS313-01 and Brix degree with an Atago refractometer (Japan, 2006) according to the method described in OIV-MA-AS2-02.

### 3.5. Analyses of Molecular Weight Distributions of Polysaccharides and Monosaccharide Composition of the Grape Pomace Extracts

The molecular weight distributions of the polysaccharides evaluated in the extracts obtained from grape pomace were determined with High-Performance Size-Exclusion Chromatography with a Refractive Index Detector (HPSEC-RID) using an Agilent Technologies 1200 Chromatograph (Santa Clara, CA, United States, 2009) following the chromatographic conditions described in Guadalupe et al. [46]. Two Shodex chromatographic columns, OHpak SB-803 HQ and OHpak SB-804 HQ (300 mm × 8 mm i.d.) with a precolumn Shodex OHpak SB-G 6B (50 mm × 6 mm i.d.). Calibration was performed with narrow dextran molecular weight standards from 410 to 5 kDa. The three different fractions were estimated to their molecular weight: high molecular weight polysaccharides (HMW P, between 700 and 100 kDa), medium molecular weight polysaccharides (MMW P, between 100 and 5 kDa) and low molecular weight polysaccharides (LMW P, <5 kDa).

The monosaccharide composition of the extracts from grape pomace was evaluated by gas chromatography–mass spectrometry (GC–MS) of their trimethylsilyl-ester O-methyl glycosyl-derivates, obtained after acidic methanolysis and derivatization, following the chromatographic conditions established in Guadalupe et al. [46]. The monosaccharide chromatogram is shown in the Supplementary Material (Figure S2). The monosaccharide data were used to determine the polysaccharide families following the method of Canalejo et al. [35]. Also, the content of non-pectic polysaccharides (NPP), total pectic polysaccharides (TPP) and the content of total families of polysaccharides (TFP) were calculated.

### 3.6. Analyses of Total Proteins, Total Polyphenols and Monomeric Phenolic Compounds of the Grape Pomaces Extracts

The extracts were diluted in water (10 mg in 10 mL) for the analyses of total proteins, total polyphenols and monomeric phenolic compounds.

The content of total proteins was determined following the Bradford Protein Assay [47] and expressed as mg of Bovine Serum Albumin (BSA) per g of extract.

The total polyphenolic compounds were determined with the reaction with Folin–Ciocalteu reaction and were expressed as mg of gallic acid (GA) per g of extract [48]. The monomeric phenolic compounds were analyzed by injection of the diluted samples in a high-performance liquid chromatograph (Agilent Technologies 1200, Waldbronn, Germany, 2010) equipped with a photodiode array detector (DAD), and following the chromatographic conditions established by Pérez-Magariño et al. [49].

### 3.7. Statistical Analyses

Analyses of variance and Fisher’s least significant difference (LSD) test were performed using the Statgraphics Centurion XVIII software (Statgraphics Technologies, Inc., The Plains, VA, USA) to determine the differences between the varietal grape pomace extracts. Principal component analysis (PCA) was carried out to study the association between variables and to determine similarities or differences between the varietal grape pomace extracts, using the RStudio program (R-Studio Inc., Version 2023.06.0, Boston, MA, USA).

## 4. Conclusions

The grape variety seems to influence the chemical composition of the extracts obtained from each pomace by-product, mainly in the types and concentrations of polysaccharides. This effect of the grape variety could be associated with cell wall thickness. The polysaccharide extracts obtained from the VER1 and PC varieties showed the highest polysaccharide purity and polysaccharide contents, but the type of polysaccharides was different in each case. The VER1 provided extracts richer in non-pectic polysaccharides, while the PC variety was the most suitable for obtaining pomace extracts richer in pectic polysaccharides. The degree of ripening may also have influenced the type of polysaccharides obtained in the extracts since it seems that the pomace from more mature grapes had a lower content of pectic polysaccharides, mainly HG and PRAG. These differences may be due to the physicochemical properties of the cell walls of the grape skins. In general, the grape pomace extracts showed a very low presence of proteins and phenolic compounds.

The yield of the grape pomace extracts also depended on the grape variety, with values ranging from 5.54% to 15.9%.

It is interesting to know the content and type of polysaccharides of the extracts obtained from the pomace of different grape varieties used in winemaking, as well as the yield and purity of each of them, in order to study the interest of their possible valorization.

These results open up a new possibility for the revalorization of grape pomace by-products to obtain polysaccharide-rich extracts, although it would be interesting to improve both the yield and the purity of the extracts obtained by studying other extraction techniques or processes.

## Figures and Tables

**Table 1:** Values of oenological parameters (±uncertainty) of the varietal grapes analyzed.

Grape Variety ^1^	Brix Degree	pH	Titratable Acidity (g of Tartaric Acid/L)	Potential Alcohol Content (% *v*/*v*)
LIG	24.2	3.3 ± 0.1	5.5 ± 0.2	14.26
VER1	23.4	3.2 ± 0.1	5.8 ± 0.2	13.68
MALV	22.7	3.7 ± 0.1	7.2 ± 0.3	13.28
SB	21.7	3.1 ± 0.1	10.2 ±0.4	12.58
RSB	21.4	3.1 ± 0.1	6.4 ± 0.3	12.36
VER2	21.2	3.5 ± 0.1	5.0 ± 0.2	12.22
AM	20.5	3.2 ± 0.1	5.9 ± 0.2	11.82
PC	19.3	3.2 ± 0.1	5.7 ± 0.2	10.99
VIU	18.2	3.0 ± 0.1	6.3 ± 0.3	10.29

^1^ Abbreviations of grape varieties in Materials and Methods section.

**Table 2:** Monosaccharide composition and polysaccharide families (mg per g of extract) of the polysaccharide extracts obtained from the different varietal grape pomace ^1^.

Compounds ^2^	LIG ^3^	VER1	MALV	SB	RSB	VER2	AM	PC	VIU	*p*-Value ^4^
**GalA**	28.94 ± 9.31 a	28.49 ± 7.46 a	45.47 ± 6.92 b	44.70 ± 5.68 b	38.91 ± 17.51 ab	53.08 ± 9.77 b	27.15 ± 0.50 a	96.05 ± 4.34 c	41.18 ± 8.83 ab	**0.0000**
**Rha**	3.26 ± 1.13 a	4.79 ± 2.44 ab	3.71 ± 0.43 a	5.26 ± 0.59 ab	3.74 ± 1.28 a	7.21 ± 0.72 b	6.43 ± 0.04 ab	11.66 ± 4.80 c	4.94 ± 1.13 ab	**0.0016**
**2-OMeXyl**	0.15 ± 0.03 a	0.23 ± 0.02 a	0.20 ± 0.01 a	0.20 ± 0.01 a	0.21 ± 0.03 a	0.24 ± 0.02 a	0.24 ± 0.00 a	0.48 ± 0.19 b	0.22 ± 0.06 a	**0.0012**
**GluA**	0.66 ± 0.14 a	1.26 ± 0.66 ab	0.78 ± 0.06 a	1.14 ± 0.12 ab	0.92 ± 0.18 ab	2.06 ± 0.18 b	1.28 ± 0.00 ab	3.46 ± 2.19 c	0.92 ± 0.23 ab	**0.0029**
**Ara**	2.37 ± 0.97 a	3.44 ± 1.07 ab	3.22 ± 0.28 ab	5.14 ± 0.23 b	2.48 ± 1.04 a	5.07 ± 0.54 b	14.07 ± 0.16 d	9.58 ± 3.55 c	3.51 ± 0.71 ab	**0.0000**
**Fuc**	0.10 ± 0.09 a	0.18 ± 0.03 abc	0.17 ± 0.02 abc	0.22 ± 0.01 bc	0.14 ± 0.03 ab	0.22 ± 0.02 bc	0.25 ± 0.00 c	0.42 ± 0.14 d	0.20 ± 0.03 bc	**0.0003**
**Gal**	16.56 ± 4.31 ab	15.87 ± 1.80 ab	24.90 ± 2.89 d	26.29 ± 0.20 d	11.02 ± 5.04 a	22.84 ± 0.15 cd	37.24 ± 0.50 e	45.03 ± 6.88 f	17.56 ± 2.81 bc	**0.0000**
**2-OMeFuc**	0.25 ± 0.04 a	0.33 ± 0.04 ab	0.33 ± 0.04 ab	0.39 ± 0.00 ab	0.25 ± 0.08 a	0.41 ± 0.02 b	0.33 ± 0.01 ab	0.74 ± 0.24 c	0.32 ± 0.03 ab	**0.0001**
**Api**	0.98 ± 0.41 ab	1.41 ± 0.30 c	1.25 ± 0.39 bc	0.69 ± 0.06 a	1.29 ± 0.02 bc	1.38 ± 0.12 bc	0.84 ± 0.03 a	1.03 ± 0.21 abc	1.04 ± 0.19 abc	**0.0178**
**KDO**	0.08 ± 0.00 a	0.18 ± 0.06 bc	1.17 ± 0.12 e	0.17 ± 0.01 abc	0.18 ± 0.05 bc	0.13 ± 0.02 ab	0.57 ± 0.06 d	0.26 ± 0.03 c	0.13 ± 0.03 ab	**0.0000**
**Gluc**	87.60 ± 15.22 b	270.58 ± 7.51 e	56.90 ± 10.45 a	99.09 ± 13.38 b	172.42 ± 25.23 d	126.54 ± 0.52 c	163.62 ± 1.40 d	147.52 ± 15.31 cd	172.42 ± 22.77 d	**0.0000**
**Man**	1.35 ± 0.75 a	1.28 ± 0.65 a	1.32 ± 0.66 a	1.90 ± 0.02 ab	0.94 ± 0.35 a	2.52 ± 0.48 b	9.12 ± 0.12 d	5.20 ± 1.24 c	1.46 ± 0.21 a	**0.0000**
**Xyl**	1.38 ± 0.54 a	1.62 ± 0.92 a	1.23 ± 0.30 a	2.33 ± 0.78 ab	1.32 ± 0.57 a	2.22 ± 0.27 a	4.42 ± 0.07 c	3.56 ± 1.61 bc	2.16 ± 0.42 a	**0.0005**
**TMS**	143.68 ± 30.68 a	329.67 ± 18.84 e	140.66 ± 16.40 a	187.52 ± 6.10 b	233.82 ± 25.69 cd	223.91 ± 11.71 bc	265.57 ± 2.78 d	324.99 ± 40.49 e	246.07 ± 25.19 cd	**0.0000**
**RG-II**	5.84 ± 1.63 a	8.63 ± 1.10 bc	11.81 ± 1.75 d	5.81 ± 0.16 a	7.70 ± 0.30 ab	8.63 ± 0.44 bc	7.98 ± 0.08 abc	10.08 ± 2.68 cd	6.89 ± 0.88 ab	**0.0004**
**MN**	1.35 ± 0.75 a	1.28 ± 0.65 a	1.32 ± 0.66 a	1.90 ± 0.02 ab	0.94 ± 0.35 a	2.52 ± 0.48 b	9.12 ± 0.12 d	5.20 ± 1.24 c	1.46 ± 0.21 a	**0.0000**
**PRAG**	20.72 ± 5.82 ab	22.55 ± 4.62 ab	29.81 ± 3.52 bc	34.52 ± 0.88 c	16.06 ± 6.67 a	33.81 ± 1.48 c	56.36 ± 0.65 d	63.44 ± 15.16 d	24.17 ± 4.77 abc	**0.0000**
**HG**	26.69 ± 9.15 ab	25.52 ± 7.19 a	42.51 ± 7.31 c	41.20 ± 5.65 bc	36.69 ± 16.83 abc	49.37 ± 9.55 c	24.16 ± 0.45 a	89.40 ± 2.19 d	38.26 ± 8.77 abc	**0.0000**
**NPP**	87.60 ± 15.22 b	270.58 ± 7.51 e	56.90 ± 10.45 a	99.09 ± 13.38 b	172.42 ± 25.23 d	126.54 ± 0.52 c	163.62 ± 1.40 d	147.52 ± 15.31 cd	172.42 ± 22.77 d	**0.0000**
**TPP**	53.25 ± 14.23 a	56.70 ± 12.88 a	84.14 ± 8.59 c	81.53 ± 6.36 bc	60.45 ± 23.21 ab	91.82 ± 11.47 c	88.50 ± 1.03 c	162.92 ± 20.02 d	69.33 ± 3.28 abc	**0.0000**
**TFP**	142.20 ± 29.54 a	328.56 ± 18.14 e	142.36 ± 16.31 a	182.52 ± 7.04 b	233.81 ± 24.47 cd	220.88 ± 11.42 c	261.24 ± 2.55 d	315.63 ± 36.57 e	243.21 ± 25.28 cd	**0.0000**
**Ara/Gal**	0.14 ± 0.05 ab	0.22 ± 0.06 c	0.13 ± 0.01 a	0.20 ± 0.01 bc	0.23 ± 0.02 c	0.22 ± 0.02 c	0.38 ± 0.00 d	0.21 ± 0.05 c	0.20 ± 0.02 bc	**0.0000**
**Rha/GalA**	0.11 ± 0.01 abc	0.16 ± 0.05 c	0.08 ± 0.00 a	0.12 ± 0.00 abc	0.10 ± 0.02 ab	0.14 ± 0.01 bc	0.24 ± 0.00 d	0.12 ± 0.04 abc	0.13 ± 0.06 abc	**0.0005**
**(Ara + Gal)/Rha**	6.02 ± 1.53 bc	5.07 ± 3.19 ab	7.62 ± 0.85 c	6.02 ± 0.60 bc	3.49 ± 0.53 a	3.89 ± 0.29 ab	7.98 ± 0.05 c	5.02 ± 1.28 ab	4.32 ± 0.54 ab	**0.0057**

^1^ Mean values ± standard deviation (n = 3). Values with different letters (a, b, c, d, e, f) in each compound or parameter indicate statistically significant differences at *p* < 0.05; ^2^ GalA: galacturonic acid; Rha: rhamnose; 2-OMeXyl: 2-O-Metilxylose; GluA: glucuronic acid; Ara: arabinose; Fuc: fucose; Gal: galactose; 2-OMeFuc: 2-O-Metilfucose; Api: apiose; KDO: 2-keto-3-deoxyoctonate ammonium salt; Gluc: glucose; Man: mannose; Xyl: xylose; TMS: total monosaccharides; RG-II: rhamnogalacturonans type II; MN: mannans; PRAG: polysaccharides rich in arabinose and galactose; HG: homogalacturonans; NPP: non-pectic polysaccharides; TPP: total pectic polysaccharides; TFP: total family polysaccharides; ^3^ grape variety abbreviations in Materials and Methods section; ^4^ statistical significative difference is indicated with *p*-values in bold (*p* < 0.05).

**Table 3:** Molecular weight distributions of polysaccharides, total proteins, total polyphenols, yield and polysaccharide purity of grape pomace extracts ^1^.

Compounds ^2^	LIG ^3^	VER1	MALV	SB	RSB	VER2	AM	PC	VIU	*p*-Value ^4^
% HMW P	41.6 ± 3.1 ab	37.7 ± 6.1 a	62.5 ± 6.3 c	47.9 ± 2.6 b	43.6 ± 7.8 ab	47.6 ± 1.5 b	58.3 ± 0.3 c	61.9 ± 0.8 c	45.6 ± 2.4 b	**0.0000**
% MMW P	nd ^5^	nd	nd	nd	nd	nd	1.4 ± 0.18	nd	nd	
% LMW P	58.4 ± 3.1 bc	62.3 ± 6.1 c	37.5 ± 6.7 a	52.1 ± 2.6 b	56.4 ± 7.8 bc	52.4 ± 1.5 b	40.3 ± 0.1 a	38.1 ± 0.8 a	54.4 ± 2.4 b	**0.0000**
Total proteins (mg BSA/g of extract)	24.5 ± 4.4 d	18.7 ± 3.7 abc	20.0 ± 4.5 bcd	18.7 ± 0.6 abc	14.5 ± 2.8 a	18.5 ± 2.6 abc	14.0 ± 0.9 a	22.9 ± 2.4 cd	16.4 ± 0.9 ab	**0.0043**
Total polyphenols (mg GA/g of extract)	35.1 ± 7.6 ef	27.9 ± 6.7 cd	19.6 ± 3.9 ab	25.9 ± 0.4 bc	36.9 ± 1.4 f	33.1 ± 2.9 def	17.2 ± 0.5 a	29.0 ± 2.9 cde	22.3 ± 1.4 abc	**0.0001**
Yield (%)	7.8 ± 0.5 ab	13.4 ± 0.9 c	6.42 ± 3.2 a	7.66 ± 0.6 ab	8.97 ± 1.7 b	9.13 ± 0.4 b	15.9. ± 0.9 d	5.54 ± 0.7 a	9.42 ± 0.4 b	**0.0000**
Polysaccharide purity (%)	14.2 ± 3.0 a	32.9 ± 1.8 e	14.2 ± 1.6 a	18.3 ± 0.7 b	23.4 ± 2.4 cd	22.1 ± 1.1 c	26.1 ± 0.3 d	31.6 ± 3.7 e	24.3 ± 2.5 cd	**0.0000**

^1^ Mean values ± standard deviation (*n* = 3). Values with different letters (a, b, c, d, e, f) in each compound or parameter indicate statistically significant differences at *p* < 0.05; ^2^ % HMW P: percentage of high molecular weight polysaccharides; % MMW P: percentage of medium molecular weight polysaccharides; % LMW P: percentage of low molecular weight polysaccharides; yield: g of extract obtained per 100 g of dry weight pomace; polysaccharide purity: g of total family polysaccharides per 100 g of extract; ^3^ grape variety abbreviations in Materials and Methods section; ^4^ statistical significative difference is indicated with *p*-values in bold (*p* < 0.05); ^5^ nd: not detected.

**Table 4:** Monomeric phenolic compounds concentration (µg/g of extract) of grape pomace extracts ^1^.

Compounds ^2^	LIG ^3^	VER1	MALV	SB	RSB	VER2	AM	PC	VIU	*p*-Value ^4^
Gallic acid	5.3 ± 1.9 c	1.7 ± 0.3 ab	n.d. ^5^	n.d.	1.4 ± 0.1 a	n.d.	2.9 ± 0.1 ab	3.2 ± 0.3 b	n.d.	**0.0024**
Protocatechuic acid	9.4 ± 1.7 a	30.1 ± 1.8 d	n.d.	10.7 ± 1.3 a	25.14 ± 0.5 c	17.3 ± 1.2 b	11.0 ± 0.6 a	19.0 ± 3.3 b	9.9 ± 0.2 a	**0.0000**
**HBA**	14.7 ± 3.0 cd	31.8 ± 1.7 g	n.d.	10.7 ± 1.3 ab	26.6 ± 0.6 f	17.3 ± 1.2 d	13.8 ± 0.7 bc	22.2 ± 3.6 e	9.9 ± 0.2 a	**0.0000**
t-caftaric acid	n.d.	148.3 ± 18.7 b	n.d.	n.d.	n.d.	56.8 ± 0.9 a	57.7 ± 0.2 a	n.d.	67.6 ± 0.5 a	**0.0000**
c-cutaric acid	n.d.	46.8 ± 4.9	n.d.	n.d.	n.d.	n.d.	n.d.	n.d.	42.9 ± 1.3	0.2583
t-cutaric acid	n.d.	51.0 ± 6.3	n.d.	n.d.	n.d.	n.d.	n.d.	n.d.	45.7 ± 2.7	0.2583
t-fertaric acid	n.d.	64.0 ± 7.5 b	n.d.	n.d.	n.d.	n.d.	n.d.	n.d.	50.7 ± 1.2 a	**0.0383**
**TE-HCA**	n.d.	310.0 ± 35.6 c	n.d.	n.d.	n.d.	56.8 ± 0.9 a	57.7 ± 0.2 a	n.d.	207.0 ± 5.7 b	**0.0000**
Catechin	342.9 ± 86.5 bc	1172.5 ± 159.0 d	137.9 ± 24.7 a	79.2 ± 3.1 a	304.0 ± 12.9 bc	396.6 ± 65.7 c	251.9 ± 7.3 b	325.1 ± 24.8 bc	112.4 ± 4.2 a	**0.0000**
Epicatechin	99.4 ± 24.5 c	206.7 ± 22.2 e	11.7 ± 0.3 a	29.8 ± 4.1 a	97.0 ± 3.6 c	71.3 ± 9.0 b	113.5 ± 2.3 cd	129.6 ± 20.7 d	22.7 ± 2.6 a	**0.0000**
Procyanidin dimer I	62.4 ± 13.1 b	387.1 ± 36.1 d	33.6 ± 1.9 a	n.d.	78.5 ± 0.8 bc	31.2 ± 0.9 a	104.6 ± 0.2 c	66.4 ± 18.6 b	97.0 ± 12.2 c	**0.0000**
Procyanidin dimer II	35.9 ± 7.6 ab	85.1 ± 14.7 d	21.3 ± 3.2 a	n.d.	56.5 ± 12.5 c	n.d.	19.5 ± 0.3 a	49.4 ± 13.8 bc	n.d.	**0.0000**
**Flavanols**	540.6 ± 124.2 b	1851.4 ± 232.2 c	204.6 ± 27.5 a	109.0 ± 7.2a	535.9 ± 15.1 b	499.2 ± 71.0 b	489.4 ± 9.0 b	570.6 ± 63.5 b	232.1 ± 10.6 a	**0.0000**
Syringetin-3-glu	13.6 ± 0.2 ab	58.1 ± 11.5 f	22.7 ± 5.7 cd	9.37 ± 1.0 a	9.6 ± 0.7 a	18.2 ± 1.1 bc	17.9 ± 0.3 bc	29.0 ± 3.4 de	32.5 ± 3.7 e	**0.0000**
Quercetin gls	13.8 ± 1.0 a	183.1 ± 28.0 e	27.5 ± 3.6 ab	21.9 ± 3.8 a	40.3 ± 1.6 bc	19.8 ± 2.3 a	22.3 ± 1.3 a	56.1 ± 9.9 cd	58.6 ± 7.7 d	**0.0000**
**Flavonols**	27.3 ± 1.2 a	241.2 ± 39.6 c	50.2 ± 9.3 a	31.2 ± 3.7 a	49.9 ± 1.4 a	38.0 ± 1.7 a	40.2 ± 1.6 a	85.1 ± 13.3 b	91.1 ± 11.4 b	**0.0000**
**Total Phenols**	582.6 ± 127.6 b	2434.5 ± 292.0 c	254.8 ± 34.7 a	150.9 ± 9.8 a	612.4 ± 15.0 b	611.3 ± 73.1 b	601.1 ± 6.6 b	677.9 ± 78.7 b	540.1 ± 26.7 b	**0.0000**

^1^ Mean values ± standard deviation (*n* = 3). Values with different letters (a, b, c, d, e, f, g) in each compound indicate statistically significant differences at *p* < 0.05; ^2^ HBA: hydroxybenzoic acids; TE-HCA: tartaric esters of hydroxycinnamic acids; glu: glucoside; gls: glycosides; ^3^ grape variety abbreviations in Materials and Methods section; ^4^ statistical significative difference is indicated with *p*-values in bold (*p* < 0.05); ^5^ n.d.: not detected.

## Data Availability

Not applicable.

## Supplementary Materials

The following supporting information can be downloaded at: https://www.mdpi.com/article/10.3390/molecules28196770/s1, Figure S1. HPSEC-RID chromatograms of all varietal extracts obtained. Abbreviations of varieties in Materials and Methods section; Figure S2. GC-MS chromatogram of the extract from Albillo Mayor grape pomace and the different identified monosaccharides. Abbreviations of compounds in Materials and Methods section.

## Sample Availability

Samples of the compounds are available from the authors.

## Abbreviations

LIG: Ligeruela; MALV: Malvasía; PC: Puesta en Cruz; RSB: Rufete Serrano Blanco; VER1: Verdejo 1; VER2: Verdejo 2; AM: Albillo Mayor; VIU: Viura; GalA: galacturonic acid; Rha: rhamnose; 2-OMeXyl: 2-O-Metilxylose; GluA: glucuronic acid; Ara: arabinose; Fuc: fucose; Gal: galactose; 2-OMeFuc: 2-O-Metilfucose; Api: apiose; KDO: 2-keto-3-deoxyoctonate ammonium salt; Gluc: glucose; Man: mannose; Xyl: xylose; TMS: total monosaccharides; RG-II: rhamnogalacturonans of type II; MN: mannans; PRAG: polysaccharides rich in arabinose and galactose; HG: homogalacturonans; NPP: non-pectic polysaccharides; TPP: total pectic polysaccharides; TFP: total family polysaccharides; HMW P: high molecular weight polysaccharides; MWM P: medium molecular weight polysaccharides; LMW P: low molecular weight polysaccharides; TP: total polyphenols; PR: total proteins; GA: gallic acid; BSA: Bovine Serum Albumin; HBA: hydroxybenzoic acids; and TE-HCA: tartaric esters of hydroxycinnamic acids.

## References

[1] Teixeira A., Baenas N., Dominguez-Perles R., Barros A., Rosa E., Moreno A.D., Garcia-Viguera C. (2014). Natural bioactive compounds from winery by-products as health promoters: A review. Int. J. Mol. Sci..

[2] Yu J., Ahmedna M. (2013). Functional components of grape pomace: Their composition, biological properties and potential applications. Int. J. Food. Sci. Technol..

[3] García-Lomillo J., González-SanJosé M.L. (2017). Applications of wine pomace in the food industry: Approaches and functions. Compr. Rev. Food Sci. Food Safety.

[4] Brenes A., Viveros A., Chamorro S., Arija I. (2016). Use of polyphenol-rich grape by-products in monogastric nutrition. A review. Anim. Feed Sci. Technol..

[5] Beres C., Costa G.N., Cabezudo I., Da Silva-James N.K., Teles A.S., Cruz A.P., Mellinger-Silva C., Tonon R.V., Cabral L.M., Freitas S.P. (2017). Towards integral utilization of grape pomace from winemaking process: A review. Waste Manag..

[6] Muhlack R.A., Potumarthi R., Jeffery D.W. (2018). Sustainable wineries through waste valorisation: A review of grape marc utilisation for value-added products. Waste Manag..

[7] Ilyas T., Chowdhary P., Chaurasia D., Gnansounou E., Pandey A., Chaturvedi P. (2021). Sustainable green processing of grape pomace for the production of value-added products: An overview. Environ. Technol. Innov..

[8] Bordiga M., Travaglia F., Locatelli M. (2019). Valorisation of grape pomace: An approach that is increasingly reaching its maturity—A review. Int. J. Food. Sci. Technol..

[9] Ky I., Lorrain B., Kolbas N., Crozier A., Teissedre P.L. (2014). Wine by-products: Phenolic characterization and antioxidant activity evaluation of grapes and grape pomaces from six different French grape varieties. Molecules.

[10] Guadalupe Z., Ayestarán B., Williams P., Doco T., Ramawat K., Mérillon J.M. (2015). Determination of must and wine polysaccharides by Gas Chromatography-Mass Spectrometry (GC-MS) and Size-Exclusion Chromatography (SEC). Polysaccharides.

[11] Ortega-Regules A., Ros-García J.M., Bautista-Ortín A.B., López-Roca J.M., Gómez-Plaza E. (2008). Changes in skin cell wall composition during the maturation of four premium wine grape varieties. J. Sci. Food Agric..

[12] Apolinar-Valiente R., Williams P., Romero-Cascales I., Gómez-Plaza E., López-Roca J.M., Ros-García J.M., Doco T. (2013). Polysaccharide composition of Monastrell red wines from four different Spanish terroirs: Effect of wine-making techniques. J. Agric. Food Chem..

[13] Apolinar-Valiente R., Romero-Cascales I., Gómez-Plaza E., López-Roca J.M., Ros-García J.M. (2015). The composition of cell walls from grape marcs is affected by grape origin and enological technique. Food Chem..

[14] Riou V., Vernhet A., Doco T., Moutounet M. (2002). Aggregation of grape seed tannins in model wine—Effect of wine polysaccharides. Food Hydrocoll..

[15] Waters E., Pellerin P., Brillouet J.M. (1994). A *Saccharomyces* mannoprotein that protects wine from protein haze. Carbohyd. Polym..

[16] Dupin I.V.S., McKinnon B.M., Ryan C., Boulay M., Markides A.J., Jones G.P., Williams P.J., Waters E.J. (2000). *Saccharomyces cerevisiae* mannoproteins that protect wine from protein haze: Their release during fermentation and lees contact and a proposal for their mechanism of action. J. Agric. Food Chem..

[17] Escot S., Feuillat M., Dulau L., Charpentier C. (2001). Release of polysaccharides by yeasts and the influence of released polysaccharides on colour stability and wine astringency. Aust. J. Grape Wine Res..

[18] Pozo-Bayón M.Á, Andújar-Ortiz I., Moreno-Arribas M.V. (2009). Volatile profile and potential of inactive dry yeast-based winemaking additives to modify the volatile composition of wines. J. Sci. Food Agric..

[19] Vidal S., Williams P., O’Neill M.A., Pellerin P. (2001). Polysaccharides from grape berry cell walls. Part I: Tissue distribution and structural characterization of the pectic polysaccharides. Carbohydr. Polym..

[20] Quijada-Morín N., Williams P., Rivas-Gonzalo J.C., Doco T., Escribano-Bailón M.T. (2014). Polyphenolic, polysaccharide and oligosaccharide composition of Tempranillo red wines and their relationship with the perceived astringency. Food Chem..

[21] Nakamura A., Yoshida R., Maeda H., Corredig M. (2006). The stabilizing behaviour of soybean soluble polysaccharide and pectin in acidified milk beverages. Int. Dairy J..

[22] Maran J.P., Swathi K., Jeevitha P., Jayalakshmi J., Ashvini G. (2015). Microwave-assisted extraction of pectic polysaccharide from waste mango peel. Carbohydr. Polym..

[23] Freitas de Oliveira C., Giordani D., Lutckemier R., Gurak P.D., Cladera-Olivera F., Ferreira Marczak L.D. (2016). Extraction of pectin from passion fruit peel assisted by ultrasound. LWT-Food Sci. Technol..

[24] Hu W., Chen S., Wu D., Zhu K., Ye X. (2021). Manosonication assisted extraction and characterization of pectin from different citrus peel wastes. Food Hydrocoll..

[25] Gharibzahedi S.M.T., Smith B., Guo Y. (2019). Ultrasound-microwave assisted extraction of pectin from fig (*Ficus carica* L.) skin: Optimization, characterization and bioactivity. Carbohydr. Polym..

[26] Lecas M., Brillouet J.M. (1994). Cell-wall composition of grape berry skins. Phytochem..

[27] Rondeau P., Gambier F., Jolibert F., Brosse N. (2013). Compositions and chemical variability of grape pomaces from French vineyard. Ind. Crops Prod..

[28] Apolinar-Valiente R., Gómez-Plaza E., Terrier N., Doco T., Ros-García J.M. (2017). The composition of cell walls from grape skin in *Vitis vinifera* intraspecific hybrids. J. Sci. Food Agric..

[29] Canalejo D., Guadalupe Z., Martínez-Lapuente L., Ayestarán B., Pérez-Magariño S., Doco T. (2022). Characterization of polysaccharide extracts recovered from different grape and winemaking products. Food Res. Int..

[30] González-Centeno M.R., Rosselló C., Simal S., Garau M.C., López F., Femenia A. (2010). Physico-chemical properties of cell wall materials obtained from ten grape varieties and their by-products: Grape pomace and stems. LWT-Food Sci. Technol..

[31] Doco T., Williams P., Pauly M., O’Neill M.A., Pellerin P. (2003). Polysaccharides from grape berry cell walls. Part II. Structural characterization of the xyloglucan polysaccharides. Carbohyd. Polym..

[32] Pinelo M., Arnous A., Meyer A.S. (2006). Upgrading of grape skins: Significance of plant cell-wall structural components and extraction techniques for phenol release. Trends Food Sci. Technol..

[33] Arnous A., Meyer A.S. (2009). Quantitative prediction of cell wall polysaccharide composition in grape (*Vitis vinifera* L.) and apple (Malus domestica) skins from acid hydrolysis monosaccharide profiles. J. Agric. Food Chem..

[34] Minjares-Fuentes R., Femenia A., Garau M.C., Candelas-Cadillo M.G., Simal S., Rossello C. (2016). Ultrasound-assisted extraction of hemicelluloses from grape pomace using response surface methodology. Carbohydr. Polym..

[35] Canalejo D., Guadalupe Z., Martínez-Lapuente L., Ayestarán B., Pérez-Magariño S. (2021). Optimization of a method to extract polysaccharides from white grape pomace by-products. Food Chem..

[36] Pérez-Magariño S., Cano-Mozo E., Bueno-Herrera M., Canalejo D., Doco T., Ayestarán B., Guadalupe Z. (2022). The effects of grape polysaccharides extracted from grape by-products on the chemical composition and sensory characteristics of white wines. Molecules.

[37] Gao Y., Fangel J.U., Willats W.G.T., Vivier M.A., Moore J.P. (2021). Differences in berry skin and pulp cell wall polysaccharides from ripe and overripe Shiraz grapes evaluated using glycan profiling reveals extensin-rich flesh. Food Chem..

[38] Garrido-Bañuelos G., Buica A., Schückel J., Zietsman A.J.J., Willats W.G.T., Moore J.P., Du Toit W.J. (2018). Investigating the relationship between grape cell wall polysaccharide composition and the extractability of phenolic compounds into Shiraz wines. Part I: Vintage and ripeness effects. Food Chem..

[39] Ortega-Regules A., Ros-García J.M., Bautista-Ortín A.B., López-Roca J.M., Gómez-Plaza E. (2008). Differences in morphology and composition of skin and pulp cell walls from grapes (*Vitis vinifera* L.): Technological implications. Eur. Food Res. Technol..

[40] Vidal S., Williams P., Doco T., Moutounet M., Pellerin P. (2003). The polysaccharides of red wine: Total fractionation and characterization. Carbohydr. Polym..

[41] Apolinar-Valiente R., Romero-Cascales I., Gómez-Plaza E., López-Roca J.M., Ros-García J.M. (2015). Cell wall compounds of red grapes skins and their grape marcs from three different winemaking techniques. Food Chem..

[42] Huang X.M., Huang H.B., Wong H.C. (2005). Cell walls of loosening skin in post-veraison grape berries lose structural polysaccharides and calcium while accumulate structural proteins. Sci. Hort..

[43] Nunan K.J., Sims I.M., Bacic A., Robinson S.P., Fincher G.B. (1998). Changes in cell wall composition during ripening of grape berries. Plant Physiol..

[44] Ortega-Regules A., Romero-Cascales I., Ros-García J.M., López-Roca J.M., Gómez-Plaza E. (2006). A first approach towards the relationship between grape skin cell-wall composition and anthocyanin extractability. Anal Chim. Acta..

[45] OIV (2016). International Organisation of Vine and Wine. Compendium of International Methods of Wine and Must Analysis.

[46] Guadalupe Z., Martínez-Pinilla O., Garrido Á., Carrillo J., Ayestarán B. (2012). Quantitative determination of wine polysaccharides by gas chromatography–mass spectrometry (GC–MS) and size exclusion chromatography (SEC). Food Chem..

[47] Bradford M.M. (1976). A rapid and sensitive method for the quantitation of microgram quantities of protein utilizing the principle of protein-dye binding. Anal. Biochem..

[48] Singleton V.L., Rossi J.A. (1965). Colorimetry of total phenolics with phosphomolibdicphos-photungstic acid reagent. Am. J. Enol. Vitic..

[49] Pérez-Magariño S., Ortega-Heras M., Cano-Mozo E. (2008). Optimization of a solid-phase extraction method using copolymer sorbents for isolation of phenolic compounds in red wines and quantification by HPLC. J. Agric. Food Chem..

