# Supplementary material for: Chemical Characterization of Polysaccharide Extracts Obtained from Pomace By-Products of Different White Grape Varieties
Source: Molecules. 2023 Sep 22;28(19):6770. doi: 10.3390/molecules28196770 (PMC10574405; doi:10.3390/molecules28196770)
Supplement: Supplementary file 1 [file molecules-28-06770-s001.zip › molecules-2614081-supplementary.pdf]

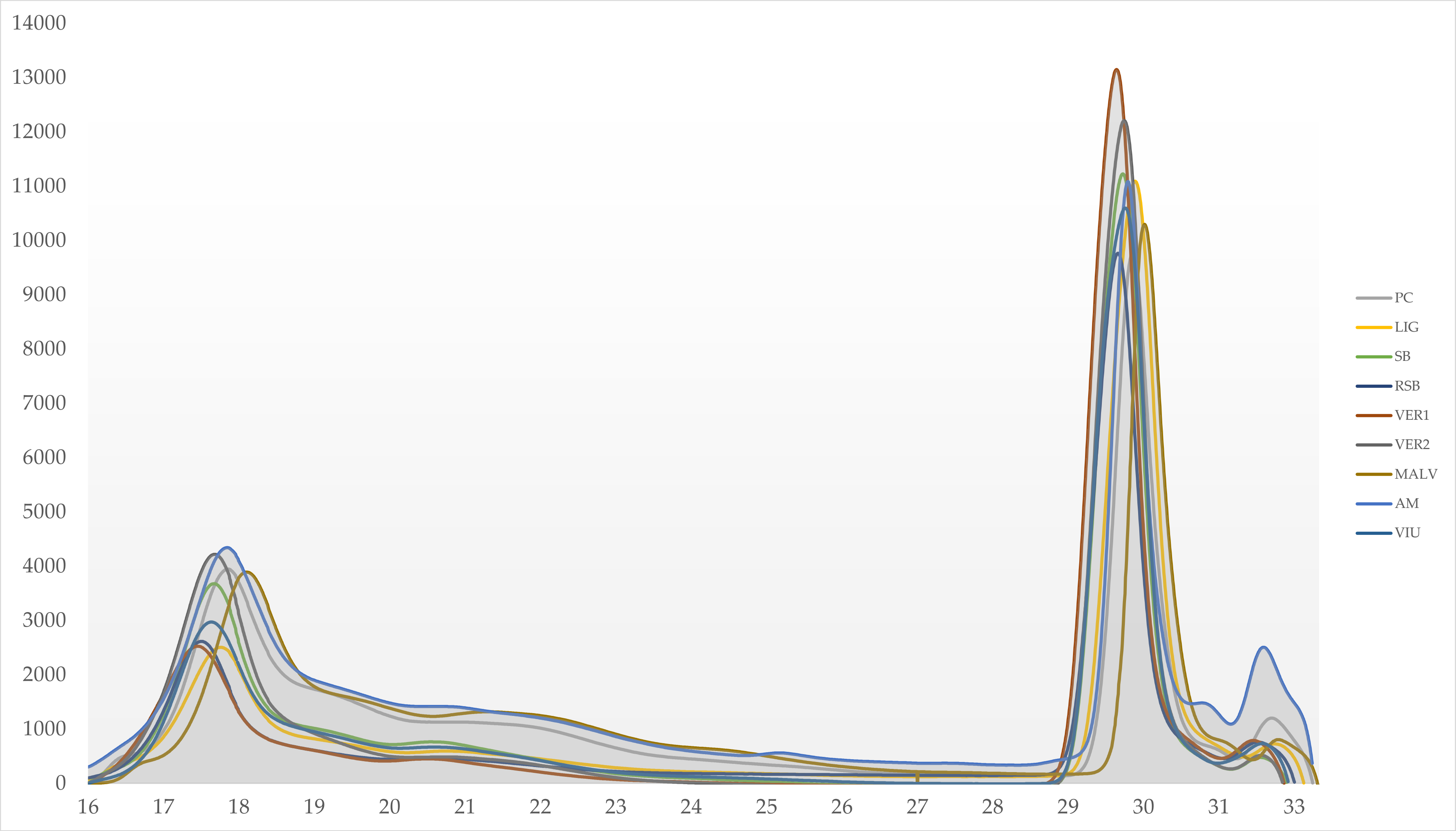


**Figure S1.** HPSEC-RID chromatograms of all varietal extracts obtained. Abbreviations of varieties in Materials and Methods section.

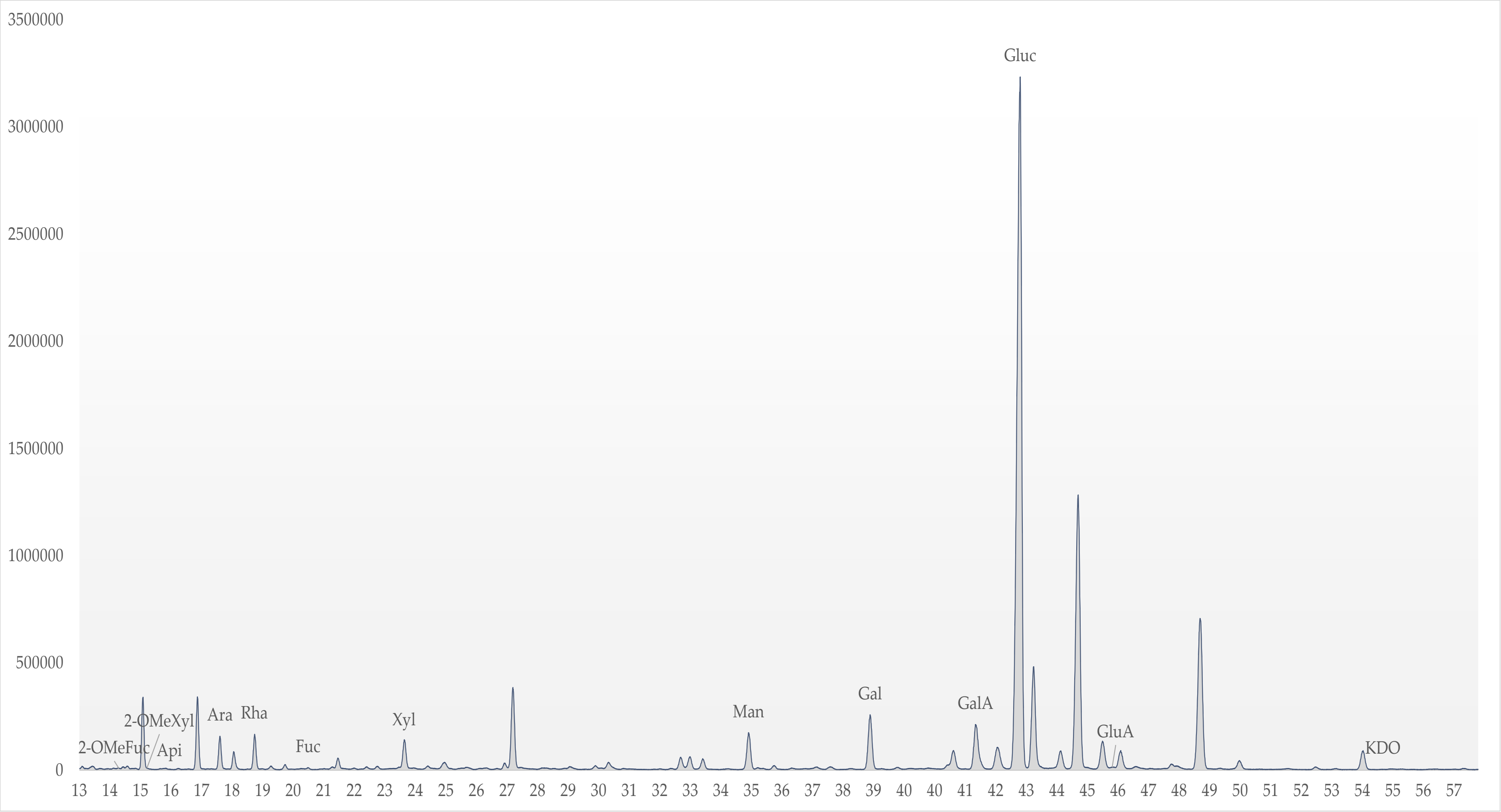


**Figure S2.** GC-MS chromatogram of the extract from Albillo Mayor grape pomace and the different identified monosaccharides. Abbreviations of compounds in Materials and Methods section.
